# High Throughput Screening of the NatureBank ‘Marine Collection’ in a *Haemonchus* Bioassay Identifies Anthelmintic Activity in Extracts from a Range of Sponges from Australian Waters

**DOI:** 10.3390/molecules26195846

**Published:** 2021-09-27

**Authors:** Aya C. Taki, Joseph J. Byrne, Abdul Jabbar, Kah Yean Lum, Sasha Hayes, Russell S. Addison, Kelsey S. Ramage, Andreas Hofmann, Merrick G. Ekins, Tao Wang, Bill C. H. Chang, Rohan A. Davis, Robin B. Gasser

**Affiliations:** 1 Department of Veterinary Biosciences, Melbourne Veterinary School, Faculty of Veterinary and Agricultural Sciences, The University of Melbourne, Parkville, VIC 3010, Australia; aya.taki@unimelb.edu.au (A.C.T.); byrnej1@unimelb.edu.au (J.J.B.); jabbara@unimelb.edu.au (A.J.); a.hofmann@structuralchemistry.org (A.H.); tao.wang1@unimelb.edu.au (T.W.); bchang@yourgene.com.tw (B.C.H.C.); 2 Griffith Institute for Drug Discovery, School of Environment and Science, Griffith University, Brisbane, QLD 4111, Australia; k.lum@griffith.edu.au (K.Y.L.); sasha.hayes2@griffithuni.edu.au (S.H.); r.addison@griffith.edu.au (R.S.A.); kelsey.ramage@griffithuni.edu.au (K.S.R.); merrick.ekins@qm.qld.gov.au (M.G.E.); r.davis@griffith.edu.au (R.A.D.); 3 Queensland Museum, South Brisbane, QLD 4101, Australia

**Keywords:** high throughput screening, *Haemonchus contortus*, parasitic nematode, anthelmintic, marine natural products, sponge

## Abstract

Widespread resistance in parasitic nematodes to most classes of anthelmintic drugs demands the discovery and development of novel compounds with distinct mechanisms of action to complement strategic or integrated parasite control programs. Products from nature—which assume a diverse ‘chemical space’—have significant potential as a source of anthelmintic compounds. In the present study, we screened a collection of extracts (*n* = 7616) derived from marine invertebrates sampled from Australian waters in a high throughput bioassay for in vitro anti-parasitic activity against the barber’s pole worm (*Haemonchus contortus*)—an economically important parasitic nematode of livestock animals. In this high throughput screen (HTS), we identified 58 active extracts that reduced larval motility by ≥70% (at 90 h), equating to an overall ‘hit rate’ of ~0.8%. Of these 58 extracts, 16 also inhibited larval development by ≥80% (at 168 h) and/or induced ‘non-wild-type’ (abnormal) larval phenotypes with reference to ‘wild-type’ (normal) larvae not exposed to extract (negative controls). Most active extracts (54 of 58) originated from sponges, three from chordates (tunicates) and one from a coral; these extracts represented 37 distinct species/taxa of 23 families. An analysis of samples by ^1^H NMR fingerprinting was utilised to dereplicate hits and to prioritise a set of 29 sponge samples for future chemical investigation. Overall, these results indicate that a range of sponge species from Australian waters represents a rich source of natural compounds with nematocidal or nematostatic properties. Our plan now is to focus on in-depth chemical investigations of the sample set prioritised herein.

## 1. Introduction

Nematodes (roundworms) of the order Strongylida (strongylids) cause some of the most significant parasitic diseases of livestock worldwide, affecting hundreds of millions of food animals (including sheep, goats, cattle and pigs), with economic losses estimated at billions of dollars per annum globally [1]. Most of these parasites are transmitted orally from contaminated pasture to the host through a direct life cycle [2]: eggs are excreted in host faeces; individual first-stage larvae (L1s) develop inside individual eggs, and then hatch (within 1 day) and develop through to the second- and third-stage larval stages (L2s and L3s) in about a week; the infective L3s are then ingested by the host, exsheath (xL3) and develop through fourth-stage larvae (L4) to dioecious adults (within 3 weeks) in the gut of the animal. The control of these nematodes has relied heavily on the use of a limited number of commercially available anthelmintic drugs. However, resistance has developed in these parasites to most classes of these drugs and is now widespread [3], and no vaccines are available against the vast majority of these worms [4]. Given the heavy reliance on anthelmintics in many parasite control programs, there is a need for the continued discovery and development of novel nematocides.

Experiences from unsuccessful combinatorial compound library screens for anthelmintic compounds [5] indicate that the discovery (hit) rate of bioactive compounds depends on the biologically-relevant ‘chemical space’ of a compound collection rather than the size of a library [6]. Unlike synthetic compounds, which are subjected to a series of structural modifications during the course of a drug discovery/development project to achieve improved drug-like or lead-like properties, natural products are produced and modified in nature over a long period of time throughout evolution. Hence, the biologically-relevant ‘chemical space’ of these products from nature is recognised to be markedly greater than in synthetic compound libraries [6]; thus, natural compounds have potential to interact with a variety of biological targets [7,8,9,10,11]. In addition, the ‘metabolite-likeness’ of natural products (i.e., their ability to act as substrates in one or more cellular systems) can facilitate the delivery of such compounds to their ultimate active sites via their binding to transporters [6]. All of these properties or features would appear to enable the drug development process, which is why some of our recent anthelmintic discovery work has assessed products from nature.

Enabling this focus are: (a) the accessibility of curated, drug-like extract-, fraction- and/or compound collections from natural sources; (b) the chemical diversity of natural products within these collections; (c) the availability of a phenotypic, whole-worm bioassays to screen these collections; and (d) the utility of advanced chromatographic, spectrometric and spectroscopic techniques for bioassay-guided fractionation and structural investigations (reviewed in [12]). This context has provided a sound basis for the identification and subsequent characterisation of anthelmintic molecules from natural sources.

Some of our discovery work has identified anthelmintic candidates in libraries of natural product-inspired compounds and extracts from plants or marine invertebrates [12,13]. For instance, in a recent study [14], we screened 2000 extracts from marine invertebrates for anthelmintic activity in a bioassay for *Haemonchus contortus* (Strongylida)—an economically significant parasitic nematode of livestock animals. Here, extracts from the sponges *Monanchora unguiculata* and *Haliclona* sp. exhibited a dose-dependent inhibition of the motility and development of larvae of *H. contortus* in vitro, and active fractions were identified by bioassay-guided fractionation of these extracts. From the active fractions from *M. unguiculata*, a pentacyclic guanidine alkaloid, fromiamycalin, was purified and shown to be an inhibitor of larval motility and development, achieving half-maximum inhibitory concentrations (IC_50_) of 4.8–39.4 µM and 0.7–26.6 µM, respectively. Investigation of the active fractions from the two *Haliclona* collections led to the identification of halaminol A and a mixture of amino alcohol lipids as active components [14]. These findings show that marine sponges are a source of anthelmintic components/molecules.

In the present study, we expand this first study to phenotypically screen a collection of extracts (*n* = 7616) derived from marine invertebrates in a high throughput bioassay for *H. contortus*. This collection has been curated by NatureBank at the Griffith Institute for Drug Discovery (GRIDD) (https://www.griffith.edu.au/institute-drug-discovery/unique-resources/naturebank) and maintained at Compounds Australia (https://www.griffith.edu.au/griffith-sciences/compounds-australia). We evaluated the nematocidal/nematostatic effects of these extracts as a basis for future work on bioassay-guided fractionation and identification/characterisation of the active molecules within these extracts.

## 2. Results and Discussion

### 2.1. High Throughput Screening Reveals 58 of 7616 Extracts with Activity on H. contortus

**Figure 1:**
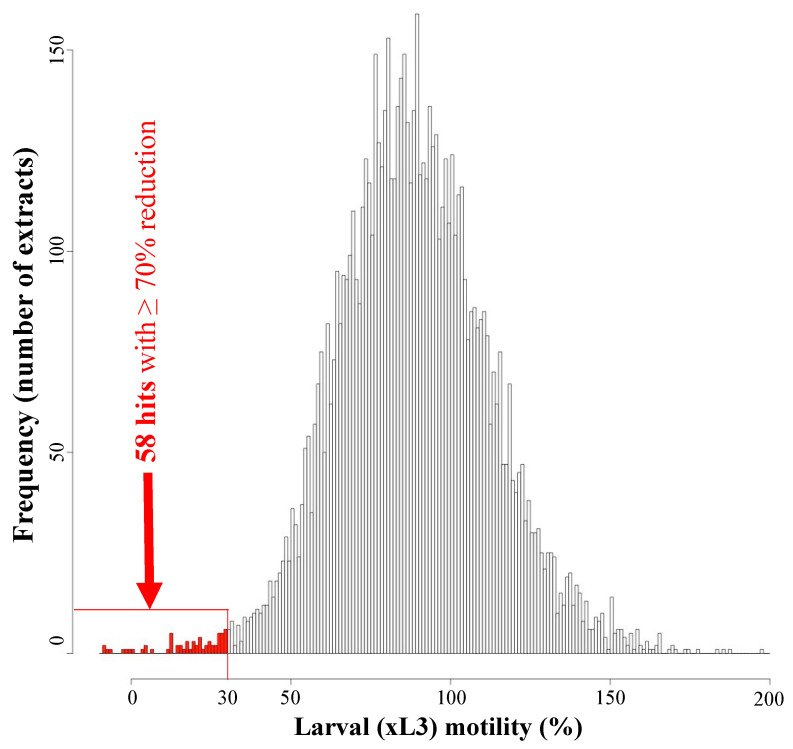
**The distribution of the numbers of extracts according to the larval motility values obtained from the high throughput screen on exsheathed third-stage larvae (xL3s) of *Haemonchus contortus.*** Of the total number of 7616 extracts individually tested at 3 µge/µL, 58 of them (~0.8%) reduced larval motility by ≥70% and were, thus, designated as ‘hits’. Motility values were normalised against those of positive- (monepantel) and negative- (no-extract/compound) controls.

Here, we screened all 7616 marine invertebrate extracts from the NatureBank collection in our high throughput assay. At 90 h, we identified 58 extracts that reduced xL3 motility by ≥70% (Table 1; Figure 1), equating to an overall “hit rate” of ~0.8%. These extracts originated from sponges (*n* = 54), chordates (tunicates; *n* = 3) and a coral, all representing 37 species/taxa of 23 distinct families (Table 1). This high proportion of marine sponge extract hits is not surprising, given that the NatureBank marine extract library is well-represented by organisms belonging to the phylum Porifera; in total, 5572 of the 7616 extracts used were derived from sponges. Marine sponges have been a significant source of novel chemistry and biology over the decades, with 9536 sponge-derived secondary metabolites currently reported, equating to ~25% of all marine natural products identified to date (http://pubs.rsc.org/marinlit/, accessed on 3 August 2021). In the past four years, the number of new sponge-derived metabolites being reported each year indicates a downward trajectory, mainly due to marine natural product chemists shifting their focus to marine microorganisms [15,16,17,18,19]. However, sponges remain an important source for the discovery of unique and bioactive natural products, and clearly warrant inclusion in drug discovery screening programs. The data reported here clearly indicate the continued importance of sponge chemistry in biodiscovery efforts.

Employing the xL3 motility cut-off of 30% (Figure 1), we identified 58 ‘hit’ candidates; the focus of such screens is to identify active compounds that substantially reduce larval motility and/or development, ultimately leading to worm destruction (if compound-effect is irreversible). However, we observed that ~35% of the extracts screened (2700 of 7616) enhanced motility beyond that of the negative control (i.e., 101% to ~200%; Figure 1). Although we cannot yet explain this finding, we suggest that some of these compounds that induce increased motility (>100%) might eventually lead to lethality over time. While we have no molecular biological knowledge of this aspect, extracts that ‘excite the worms to exhaustion’ might be candidates for further explorations. Nonetheless, here, the focus was on extracts that reduced larval motility and development.

**Figure 2:**
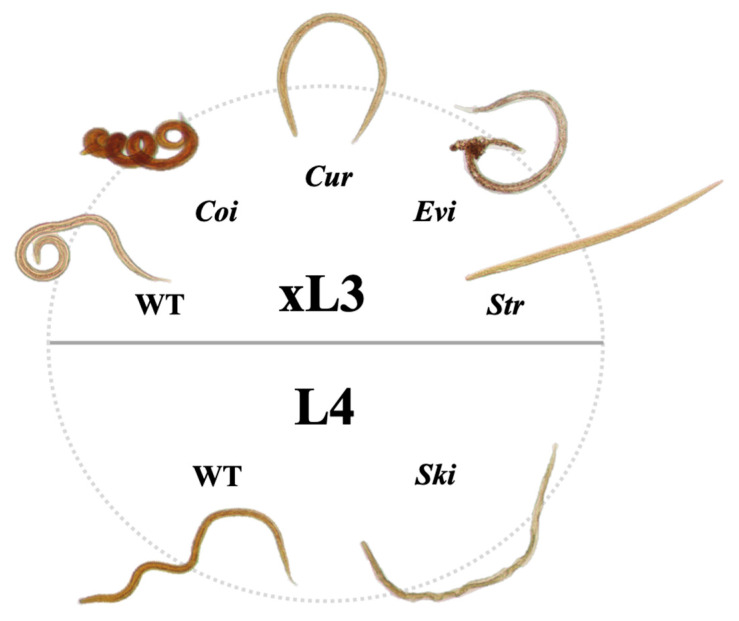
**Schematic summary of distinct larval phenotypes of exsheathed third-stage (xL3) or fourth-stage (L4) larvae of *Haemonchus contortus* observed in this study**. For the xL3 stage, wild-type (WT; ‘normal’), coiled (*Coi*), curved (*Cur*), eviscerated (*Evi*) and straight (*Str*) phenotypes. For the L4 stage, wild-type (WT; ‘normal’) and skinny (*Ski*) phenotypes. A WT phenotype displays motility consistent with that seen in negative control wells (containing LB* medium with ≤1.2% DMSO) at a respective time point (i.e., 90 h for xL3 and 168 h for L4). *Coi*, *Cur*, *Evi*, *Str* and *Ski* phenotypes were usually immotile. The lengths of wild-type xL3s and L4s cultured in vitro (without extract or compound) for 90 h and 168 h are ~600–650 µm and 700–750 µm, respectively.

Some ‘hit’ extracts (codes) from sponges *Agelas axifera* (NB028803), *Ceratopsion clavatum* (NB6018007), *Coscinoderma mathewsi* (NB6013853 and NB5866375), *Echinochalina* (*Protophlitaspongia*) sp. (NB6005201)*, Phyllospongia bergquistae* (NB5376298, NB6005361 and NB5818101), *Phyllospongia papyracea* (NB6017543 and NB010981), *Polyfibrospongia* *flabellifera* (NB6013898) or *Rhabdastrella globostellata* (NB023362) inhibited xL3 motility by ≥90% at 90 h (Table 1). At 168 h, 16 of these 58 extracts also inhibited larval development by ≥80% and/or induced ‘non-wild-type’ (abnormal) larval phenotypes with reference to ‘wild-type’ (normal) larvae exposed to DMSO only (negative control) (Table 1). Marked differences in larval morphology were seen both within and between wells, with curved (*Cur*) and/or coiled (*Coi*) phenotypes predominating when larval developmental inhibition was ≥80% at 168 h of exposure (Table 1; Figure 2). Four of these 16 extracts that inhibited both xL3 motility by ≥90% (90 h) and L4 development by ≥80% (168 h) were from *Agelas axifera* (NB028803), *Echinochalina* (*Protophlitaspongia*) sp. (NB6005201), *Phyllospongia bergquistae* (NB5818101) and *Polyfibrospongia* *flabellifera* (NB6013898) (Table 1).

### 2.2. NMR Fingerprints of Extracts

While the screening clearly identified that extracts from multiple sponge species should be prioritised for further chemical investigations, our past HTS biodiscovery experience has indicated that hits representing congeners typically contain similar or the same chemistry, albeit at varying concentrations. Thus, we subjected all hit extracts to ^1^H NMR analysis prior to large-scale extraction and bioassay-guided fractionation studies, with the exception of extracts NB5818080 and NB008063, for which insufficient amounts of material were available. Given that numerous taxonomic clusters were represented (Table 1), we hoped that ^1^H NMR fingerprinting of individual hit extracts would allow us to rapidly dereplicate the hit list and prioritise samples for detailed chemical investigations.

**Figure 3:**
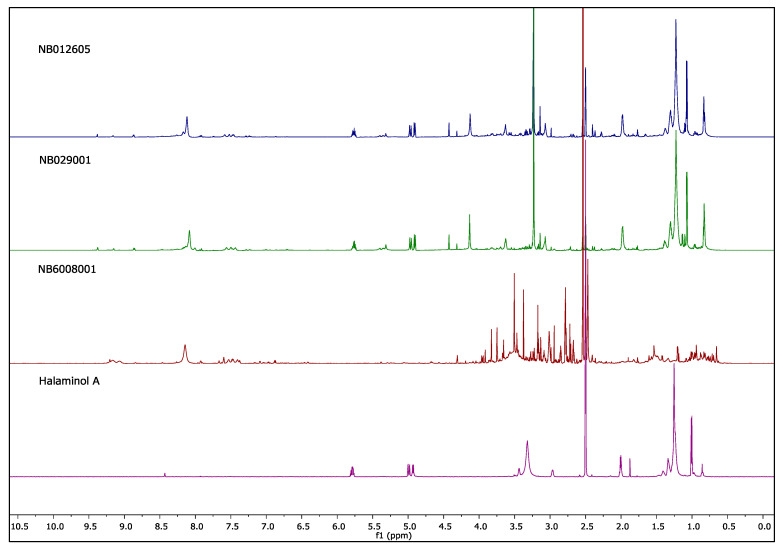
**^1^H NMR (800 MHz) spectra of *Haliclona* extracts and halaminol A in DMSO-*d*_6_.** An example of the dereplication of three extracts (NB012605, NB029001 and NB6008001; Table 1) from species of *Haliclona.*

Following our preliminary ^1^H NMR data analysis, some chemistry replicates were evident in the nine taxonomic clusters, which included the genera *Callyspongia* (*n* = 4), *Clathria* (2), *Coscinoderma* (9), *Haliclona* (3), *Oceanapia* (2), *Phyllospongia* (11), *Psammocinia* (2), *Rhabdastrella* (3) and *Didemnum* (2). The ^1^H NMR spectra clustering of all hit extracts are presented in the Supplementary Materials (S1–S13). For example, the ^1^H NMR fingerprinting of the three *Haliclona* sp. extracts in DMSO-*d*_6_ (Figure 3) showed that extracts NB012605 and NB029001 have similar chemical profiles. Based on a previous anthelmintic evaluation of *Haliclona* sp. [14], the signals and multiplicities seen at *δ*_H_ 5.77, 4.97, 4.91, 1.98, 1.38–1.23 and 1.07 indicate the presence of halaminol A (Figure 3) and/or its derivatives in both extracts; thus, it was postulated that halaminols are likely the molecules responsible for the inhibitory activities and induction of abnormal phenotypes linked to these hit extracts. The absence of halaminol signals from the ^1^H NMR spectrum of the extract NB6008001, along with the presence of different signals indicate that other chemotypes might be responsible for the anti-nematode activity, which led us to prioritise only this sample from the *Haliclona* cluster for future chemical investigation.

Overall, ^1^H NMR fingerprints of extracts derived from the same taxonomic cluster, including *Coscinoderma mathewsi*, *Oceanapia* sp., *Phyllospongia* sp. and *Rhabdastrella globostellata* showed essentially super-imposable spectra, with only four exceptions: species of *Callyspongia*, *Clathria*, *Psammocinia* and *Didemnum* (cf. Supplementary Materials). As a result of this dereplication work, all singleton hits (*n* = 19) will be pursued in follow-up chemical investigations while only one or two samples (12 in total) from the nine taxonomy clusters will proceed to future bioassay-guided fractionation studies.

In conclusion, we show that a range of sponges collected from Australian waters represents a rich source of natural compounds with nematocidal/nematostatic activities. Some species of *Agelas, Echinochalina* (or *Protophlitaspongia*), *Phyllospongia* and *Polyfibrospongia*, in particular, appear to contain compounds that are potently active against early larval stages of *H. contortus*, and warrant further investigation. Whether the anti-nematode effects seen here relate to molecules produced by the sponges themselves or by the microbes associated with these sponges (such as symbionts—mutualists, commensals and/or parasites) remains to be established. Our next step will be to characterise pure nematocidal/nematostatic molecules, identify where and how these molecules are produced in the sponge, assess whether they are toxic to mammalian cells and establish their mechanism(s) of action in nematodes.

## 3. Materials and Methods

### 3.1. Marine Extract Collection

The collection of 7616 marine-derived extracts was purchased from NatureBank, Griffith Institute for Drug Discovery (GRIDD; https://www.griffith.edu.au/institute-drug-discovery), Queensland, Australia. These extracts are derived from marine invertebrate samples collected from Australian waters—the biota represent 277 families and 17 distinct phyla. At GRIDD, freeze-dried marine materials were processed to produce extracts [21,22]. Individual extracts were solubilised in dimethyl sulfoxide (DMSO; Sigma-Aldrich, St Louis, MO, USA) and stocks of 500 µge/µL prepared. This concentration unit, µge/µL, relates to: (i) the amount of dry biota material used for extraction and (ii) the amount of DMSO in which the extract was dissolved, prior to screening. This means that a 300 mg-equivalent (mge) represents an extract derived from 300 mg of dry material from a marine invertebrate; when this extract is dissolved in 0.6 mL of solvent (i.e., DMSO), the final stock solution concentration is 500 µge/µL.

### 3.2. Preparation of Haemonchus contortus Larvae

Third-stage larvae (L3s) of *H. contortus* (Haecon-5 strain) were produced and stored using a well-established protocol [20]—approved by the animal ethics committee of the University of Melbourne (permit no. 1714374). On the day of screening, L3s were exsheathed and sterilised by incubation in 0.15% (*v/v*) sodium hypochlorite (NaClO) at 38 °C for 20 min [20] and then immediately washed five times in sterile saline by centrifugation at 500× *g* (5 min) at room temperature (22–24 °C). After the last wash, exsheathed L3s (xL3s) were suspended in Luria Bertani broth (LB) containing 100 IU/mL of penicillin, 100 µg/mL of streptomycin and 0.25 µg/mL of amphotericin B (Fungizone; Thermo Fisher Scientific, Waltham, MA, USA)—designated LB*.

### 3.3. Screening of Extracts for Larval Motility Reduction (90 h) and Developmental/Morphological Alterations (168 h)

Extracts were individually diluted to 3 μge/μL in LB* containing ≤1.2% (*v*/*v*) DMSO and then dispensed in 20 µL into the wells of sterile 384-well flat-bottom microtitre plates (cat. no. 3680; Corning, Corning, NY, USA); at least eight wells represented negative controls (with LB* + ≤1.2% DMSO) and four wells containing each monepantel (Zolvix; Elanco, Greenfield, IN, USA), moxidectin (Cydectin; Virbac, Carros, France), monepantel/abamectin (Zolvix Plus; Elanco, Greenfield, IN, USA) and compound MIPS-0018666 (abbreviated here as M-666; see [23]) as known positive controls (20 µM) [20]. Following the dilution and dispensing of extracts into plates, 80 xL3s in 20 μL of LB* were added to each well; this number of larvae per well was optimised in well-controlled experiments by serial titration. Plates were incubated at 38 °C, 10% (*v*/*v*) CO_2_ and a relative humidity of >90%.

After 90 h of incubation of xL3s with individual extracts (3 μge/μL), larval motility was measured for 15 min in each well of each plate by infrared light beam-interference [24] employing a WMicroTracker ONE instrument (Phylumtech, Sunchales, Santa Fe, Argentina) using the mode 1_threshold-average setting (described in the user manual for this instrument). Raw data captured were normalised using measurements for the positive (M-666) and negative (LB* + ≤1.2% DMSO) controls, in order to remove plate-to-plate variation by calculating the percentage of motility using the program GraphPad Prism v.9.1.0 (GraphPad Software, San Diego, CA, USA). A compound was recorded as a “hit” if it reduced larval motility by ≥70%, and primary hits were re-screened. The performance of this assay was continually monitored using the Z’-factor [25], calculated using data for the negative (DMSO) and the M-666 controls from individual plates. Assays with a ‘sound’ performance achieve a Z’-factor of 0.5 to 1; the present assay consistently achieved ≥0.8. We also measured the signal to background (S/B) ratio [26] using data from the same control wells; this ratio was consistently >200.

Following the measurement of xL3 motility in the WMicroTracker ONE instrument, plates were returned to the incubator (same conditions) for additional 78 h. Then, larvae in individual wells were fixed with 40 µL of 1% iodine and microscopically examined (using an M80 light microscope; Leica, Wetzlar, Germany) at 60-times magnification to assess their development (based on the presence/absence of a well-developed pharynx; reference [27]) and morphology (phenotype). At 168 h, xL3s exposed to LB* with ≤1.2% DMSO are expected to reach the L4 stage in vitro within 168 h [28].

### 3.4. ^1^H NMR Fingerprinting of Hit Extracts

NMR spectra were recorded at 25 °C on an AVANCE III HD 800 MHz NMR spectrometer (Bruker, Billerica, MA, USA), equipped with a cryoprobe. Individual extracts were dissolved in 180 µL of DMSO-*d*_6_ and run in a 3 mm NMR tube. For each sample, the following parameters were applied, pw = 30°, p1 = 9.250 µs, d1 = 1 s, at = 2.04 s, sw = 20.03 ppm, nt = 64 scans [22]. The ^1^H chemical shifts were referenced to the solvent peak for DMSO-*d*_6_ at δ_H_ 2.50. NMR data were processed using MestReNova software v.11.0.4 (Mestrelab Research, Santiago de Compostela, Spain).

## Figures and Tables

**Table 1:** **The 58 ‘hit’ extracts identified in the primary screen with in vitro activity against exsheathed third-stage larvae (xL3s) of *Haemonchus contortus***. Information about the extracts, including code and source organism (species and family/group) used for screening, is listed. The results from the screen include xL3 motility inhibition (threshold: ≥70%) at 90 h; developmental inhibition (threshold: ≥80%) of fourth-stage larvae (L4) at 168 h; and the larval phenotypes observed at 168 h. Larval phenotypes observed were coiled (*Coi*), curved (*Cur*), eviscerated (*Evi*), skinny (*Ski*) or straight (*Str*) (Figure 2; cf. [20]). Shaded are extracts that inhibited larval motility by ≥90% (90 h) and development by ≥80% (168 h) in the assay.

NatureBank extract code	Species	Family (group*)	xL3 motility inhibition at 90 h	L4 development inhibition at 168 h	Abnormal phenotype detected (%) at 168 h
NB5866465	*Acanthophora muscoides*	Rhodomelaceae (s)	71	No	nd
NB028803	*Agelas axifera*	Agelasidae (s)	96	Yes	*Cur* (80)
NB029537	*Callyspongia (Callyspongia)* sp.	Callyspongiidae (s)	76	No	nd
NB015513	*Callyspongia (Callyspongia)* sp.	Callyspongiidae (s)	75	No	nd
NB007753	*Callyspongia (Euplacella)* sp.	Callyspongiidae (s)	79	No	nd
NB6016992	*Callyspongia (Toxachalina)* sp.	Callyspongiidae (s)	82	Yes	*Cur* (95)
NB6018007	*Ceratopsion clavatum*	Raspailiidae (s)	107	No	nd
NB6004722	*Chalinula* sp.	Chalinidae (s)	75	No	nd
NB031644	*Cinachyrella (Raphidotethya) enigmatica*	Tetillidae (s)	72	Yes	*Cur* (40), *Coi* (20)
NB6018049	*Citronia* sp.	Dysideidae (s)	70	No	*Ski* (60), *Cur* (40)
NB6014898	*Clathria (Thalysias) reinwardti*	Microcionidae (s)	72	No	nd
NB6020295	*Clathria* sp.	Microcionidae (s)	73	No	nd
NB6013853	*Coscinoderma mathewsi*	Spongiidae (s)	102	No	*Cur* (30), *Evi* (25)
NB5866375	*Coscinoderma mathewsi*	Spongiidae (s)	97	No	nd
NB6007999	*Coscinoderma mathewsi*	Spongiidae (s)	88	Yes	nd
NB6013552	*Coscinoderma mathewsi*	Spongiidae (s)	87	No	*Cur* (40), *Evi* (10)
NB6008047	*Coscinoderma mathewsi*	Spongiidae (s)	84	No	nd
NB6009651	*Coscinoderma mathewsi*	Spongiidae (s)	79	No	nd
NB5866277	*Coscinoderma mathewsi*	Spongiidae (s)	72	No	nd
NB6008378	*Coscinoderma mathewsi*	Spongiidae (s)	71	No	nd
NB6009654	*Coscinoderma mathewsi*	Spongiidae (s)	70	Yes	nd
NB5379207	*Cymbastela coralliophila*	Axinellidae (s)	80	No	*Ski* (90)
NB6009659	*Desmacella* sp.	Desmacellidae (s)	83	No	*Cur* (70), *Evi* (10)
NB6005201	*Echinochalina (Protophlitaspongia)* sp.	Microcionidae (s)	100	Yes	*Cur* (95)
NB6020433	*Endectyon* sp.	Raspailiidae (s)	75	No	nd
NB6003967	*Erylus amissus*	Geodiidae (s)	85	No	*Cur* (50)
NB5866232	*Fascaplysinopsis reticulata*	Thorectidae (s)	73	No	*Ski* (95)
NB6018006	*Gelliodes* sp.	Niphatidae (s)	71	No	nd
NB012605	*Haliclona (Haliclona)* sp.	Chalinidae (s)	85	Yes	*Str* (90), *Cur* (10)
NB029001	*Haliclona (Reniera)* sp.	Chalinidae (s)	83	Yes	*Str* (99)
NB6008001	*Haliclona (Reniera)* sp.	Chalinidae (s)	72	No	nd
NB031634	*Ianthella flabelliformis*	Ianthellidae (s)	88	No	nd
NB5820466	*Leucetta microraphis*	Leucettidae (s)	78	Yes	*Coi* (80)
NB6009581	*Oceanapia* sp.	Phloeodictyidae (s)	80	Yes	*Cur* (30), *Coi* (30)
NB6009479	*Oceanapia* sp.	Phloeodictyidae (s)	74	No	*Cur* (90), *Coi* (20), *Evi* (10)
NB027467	*Petromica (Chaladesma) pacifica*	Desmanthidae (s)	77	No	*Ski* (60), *Cur* (20)
NB6020712	*Phyllospongia foliascens*	Thorectidae (s)	81	Yes	*Cur* (80)
NB6021239	*Phyllospongia foliascens*	Thorectidae (s)	72	No	*Cur* (80)
NB5376298	*Phyllospongia bergquistae*	Thorectidae (s)	108	No	*Cur* (30), *Evi* (20), *Coi* (20)
NB6005361	*Phyllospongia bergquistae*	Thorectidae (s)	107	No	*Cur* (30), *Evi* (10)
NB5818101	*Phyllospongia bergquistae*	Thorectidae (s)	101	Yes	nd
NB028821	*Phyllospongia bergquistae*	Thorectidae (s)	88	Yes	*Cur* (100)
NB2434682	*Phyllospongia bergquistae*	Thorectidae (s)	79	Yes	nd
NB6017543	*Phyllospongia bergquistae*	Thorectidae (s)	108	No	*Cur* (30), *Evi* (10)
NB010981	*Phyllospongia papyracea*	Thorectidae (s)	93	No	*Cur* (60)
NB5818080	*Phyllospongia papyracea*	Thorectidae (s)	88	No	nd
NB6017542	*Phyllospongia papyracea*	Thorectidae (s)	85	No	*Cur* (30), *Coi* (20)
NB6013898	*Polyfibrospongia* *flabellifera*	Thorectidae (s)	99	Yes	*Cur* (60)
NB5867103	*Psammocinia halmiformis*	Irciniidae (s)	75	No	nd
NB6005306	*Psammocinia* sp.	Irciniidae (s)	84	Yes	*Cur* (95)
NB008063	*Pseudoceratina* sp.	Pseudoceratinidae (s)	80	No	nd
NB023362	*Rhabdastrella globostellata*	Ancorinidae (s)	95	No	*Ski* (90)
NB6008040	*Rhabdastrella globostellata*	Ancorinidae (s)	81	No	nd
NB5818959	*Rhabdastrella globostellata*	Ancorinidae (s)	78	No	*Ski* (90)
NB6018071	*Didemnum molle*	Didemnidae (t)	71	No	nd
NB5867348	*Didemnum perplexum*	Didemnidae (t)	73	No	nd
NB6021174	*Polycarpa aurata*	Styelidae (t)	73	No	nd
NB029740	*Sarcophyton cherbonnieri*	Alcyoniidae (c)	80	No	nd

* sponge (s); tunicate (t); coral (c). Not detected (nd).

## Data Availability

The data presented in this study are available in the article.

## Supplementary Materials

The following materials are available: stacked and clustered ^1^H NMR data for hit extracts.
