# Supplementary material for: High Throughput Screening of the NatureBank ‘Marine Collection’ in a Haemonchus Bioassay Identifies Anthelmintic Activity in Extracts from a Range of Sponges from Australian Waters
Source: Molecules. 2021 Sep 27;26(19):5846. doi: 10.3390/molecules26195846 (PMC8512444; doi:10.3390/molecules26195846)
Supplement: Supplementary file 1 [file molecules-26-05846-s001.zip › molecules-1349028-supplementary.pdf]

Supplementary Materials

High Throughput Screening of the NatureBank ‘Marine Collection’ in a *Haemonchus* Bioassay Identifies Anthelmintic Activity in Extracts from a Range of Sponges from Australian Waters

Aya C. Taki [1], Joseph J. Byrne [1], Abdul Jabbar [1], Kah Yean Lum[2], Sasha Hayes [2], Russell S. Addison [2], Kelsey S. Ramage[2], Andreas Hofmann [1], Merrick G. Ekins [2,3], Tao Wang [1], Bill C. H. Chang [1], Rohan A. Davis [2] and Robin B. Gasser [1*]

[1] Department of Veterinary Biosciences, Melbourne Veterinary School, Faculty of Veterinary and Agricultural Sciences, The University of Melbourne, Parkville, Victoria 3010, Australia; aya.taki@unimelb.edu.au (A.C.T.); byrnej1@unimelb.edu.au (J.J.B.); jabbara@unimelb.edu.au (A.J.); a.hofmann@structuralchemistry.org (A.H.); tao.wang1@unimelb.edu.au (T.W.); bchang@yourgene.com.tw (B.C.H.C.)

[2] Griffith Institute for Drug Discovery, School of Environment and Science, Griffith University, Brisbane, Queensland 4111, Australia; k.lum@griffith.edu.au (K.Y.L.); sasha.hayes2@griffithuni.edu.au (S.H.); r.addison@griffith.edu.au (R.S.A.); kelsey.ramage@griffithuni.edu.au (K.S.R.); r.davis@griffith.edu.au (R.A.D.)

[3] Queensland Museum, South Brisbane, Queensland 4101, Australia; merrick.ekins@qm.qld.gov.au (M.G.E.).

* Correspondence: robinbg@unimelb.edu.au (R.B.G.)

Contents

S1 ^{1}H NMR (800 MHz) Spectra of *Callyspongia* sp. Extracts in DMSO-d_6

S2 ^{1}H NMR (800 MHz) Spectra of *Clathria* sp. Extracts in DMSO-d_6

S3 ^{1}H NMR (800 MHz) Spectra of *Coscinoderma mathewsi* Extracts in DMSO-d_6

S4 ^{1}H NMR (800 MHz) Spectra of *Haliclona* sp. Extracts in DMSO-d_6

S5 ^{1}H NMR (800 MHz) Spectra of *Oceanapia* sp. Extracts in DMSO-d_6

S6 ^{1}H NMR (800 MHz) Spectra of *Phyllospongia* sp. Extracts in DMSO-d_6

S7 ^{1}H NMR (800 MHz) Spectra of *Psammocinia* sp. Extracts in DMSO-d_6

S8 ^{1}H NMR (800 MHz) Spectra of *Rhabdastrella globostellata* Extracts in DMSO-d_6

S9 ^{1}H NMR (800 MHz) Spectra of *Didemnum* sp. Extracts in DMSO-d_6

S10 ^{1}H NMR (800 MHz) Spectra of *Acanthophora muscoides*, *Agelas axifera*, *Ceratopsion clavatum*, *Chalinula* sp. and *Cinachyrella (Raphidotethya) enigmatica* Extracts in DMSO-d_6

S11 ^{1}H NMR (800 MHz) Spectra of *Citronia* sp., *Cymbastela coralliophila*, *Desmacella* sp., *Echinochalina (Protophlitaspongia)* sp. and *Endectyon* sp. Extracts in DMSO-d_6

S12 ^{1}H NMR (800 MHz) Spectra of *Erylus amissus*, *Fascaplysinopsis reticulata*, *Gelliodes* sp., *Ianthella flabelliformis* and *Leucetta microraphis* Extracts in DMSO-d_6

S13 ^{1}H NMR (800 MHz) Spectra of *Petromica (Chaladesma) pacifica*, *Polyfibrospongia flabellifera*, *Polycarpa aurata* and *Sarcophyton cherbonnieri* Extracts in DMSO-d_6

S1 ^{1}H NMR (800 MHz) Spectra of *Callyspongia* sp. Extracts in DMSO-d_6[a]

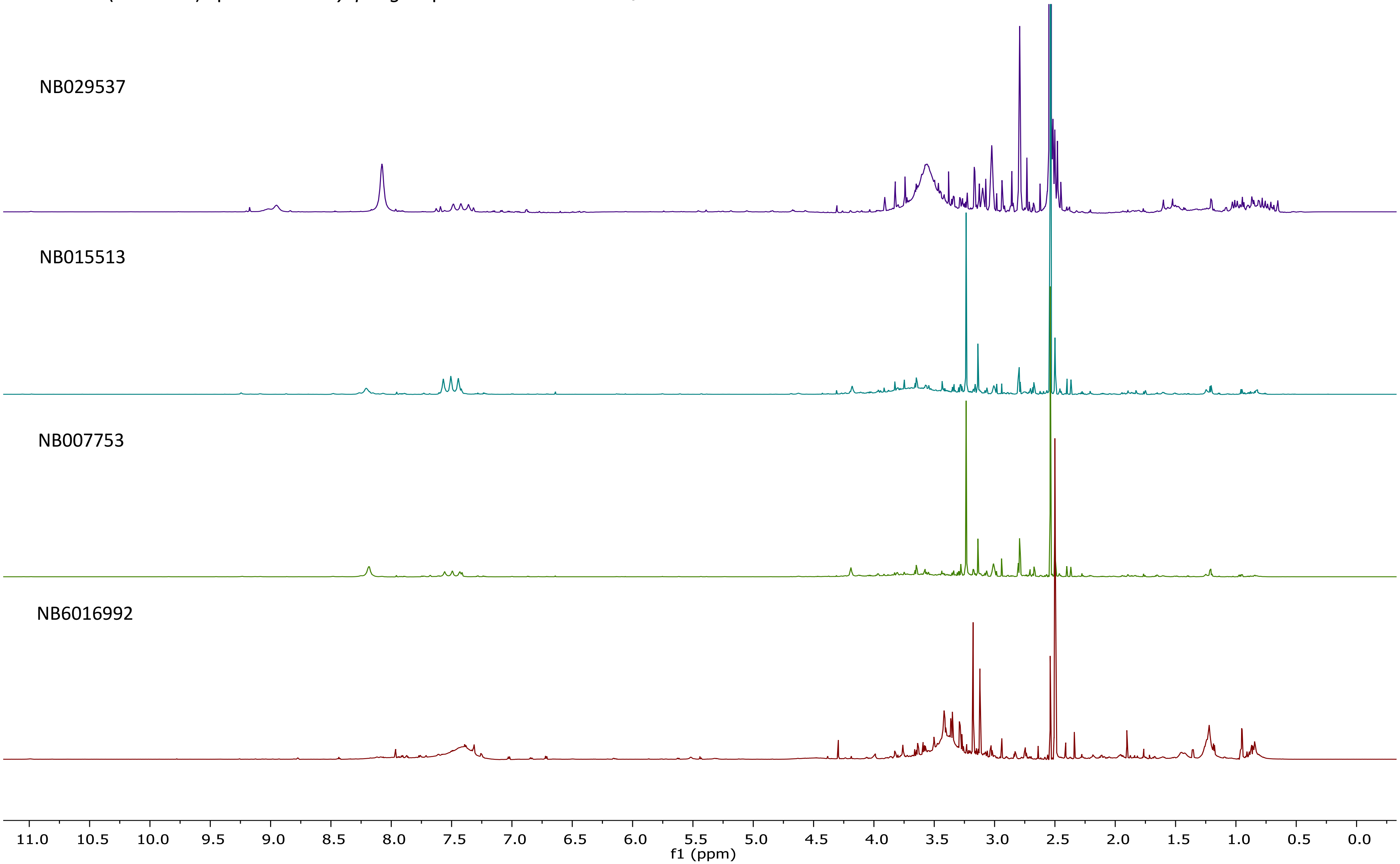

[a]Singlet at δ_H 2.54 represents DMSO residual peak

S2 ^{1}H NMR (800 MHz) Spectra of *Clathria* sp. Extracts in DMSO-d_6[a]

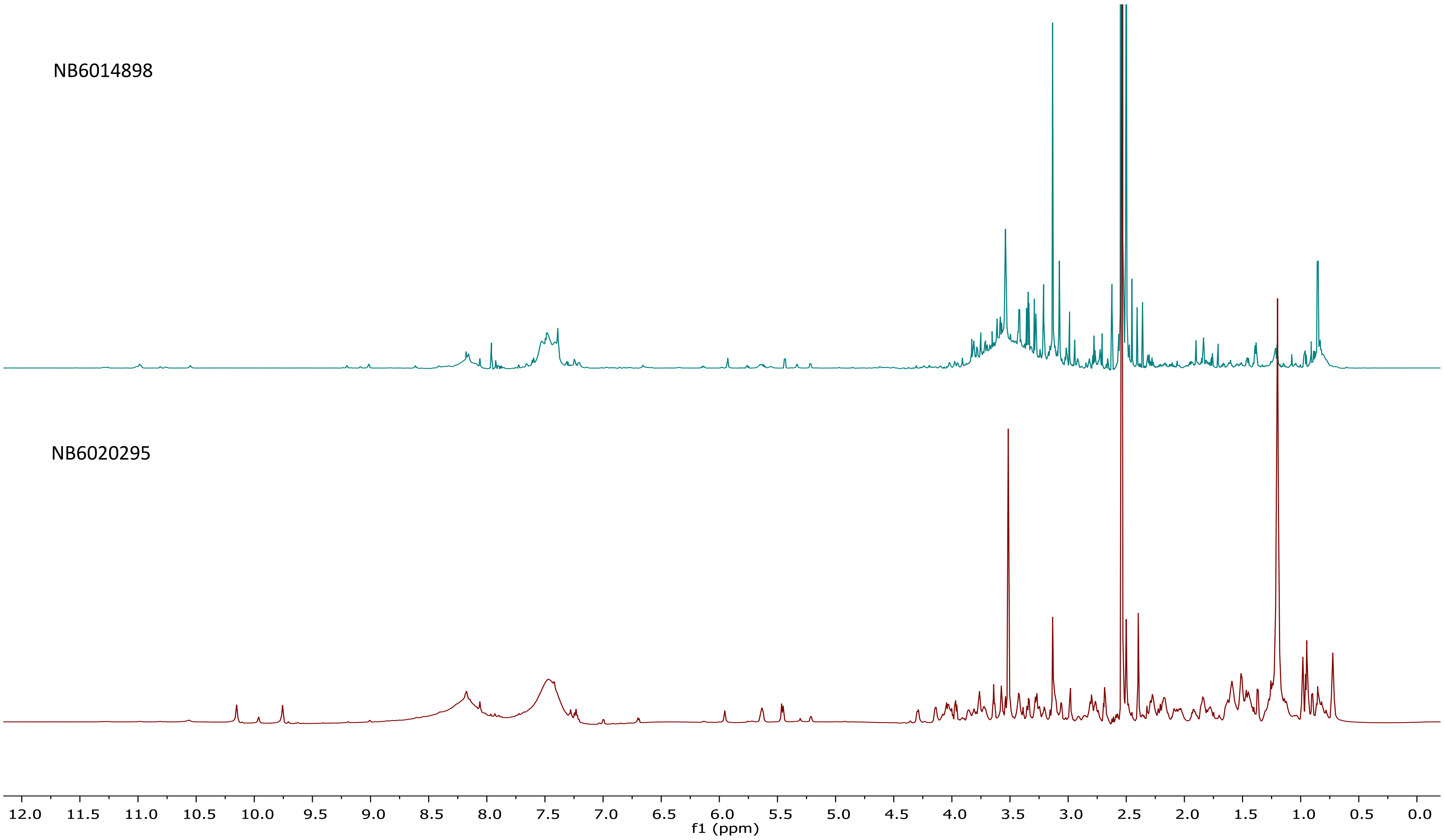

[a]Singlet at δ_H 2.54 represents DMSO residual peak

S3 ^{1}H NMR (800 MHz) Spectra of *Coscinoderma mathewsi* Extracts in DMSO-d_6[a]

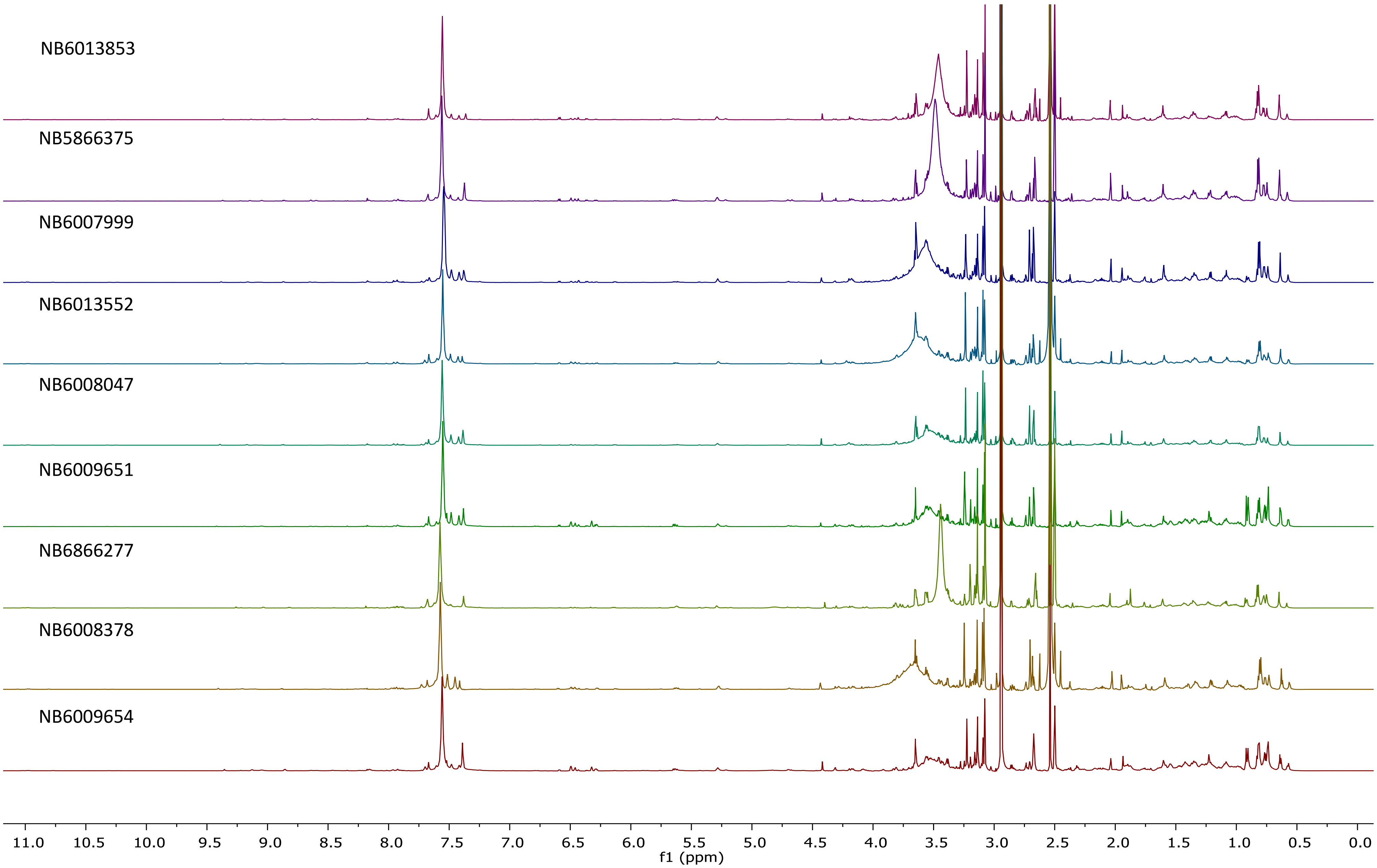

[a]Singlet at δ_H 2.54 represents DMSO residual peak

S4 ^{1}H NMR (800 MHz) Spectra of *Haliclona* sp. Extracts in DMSO-d_6[a]

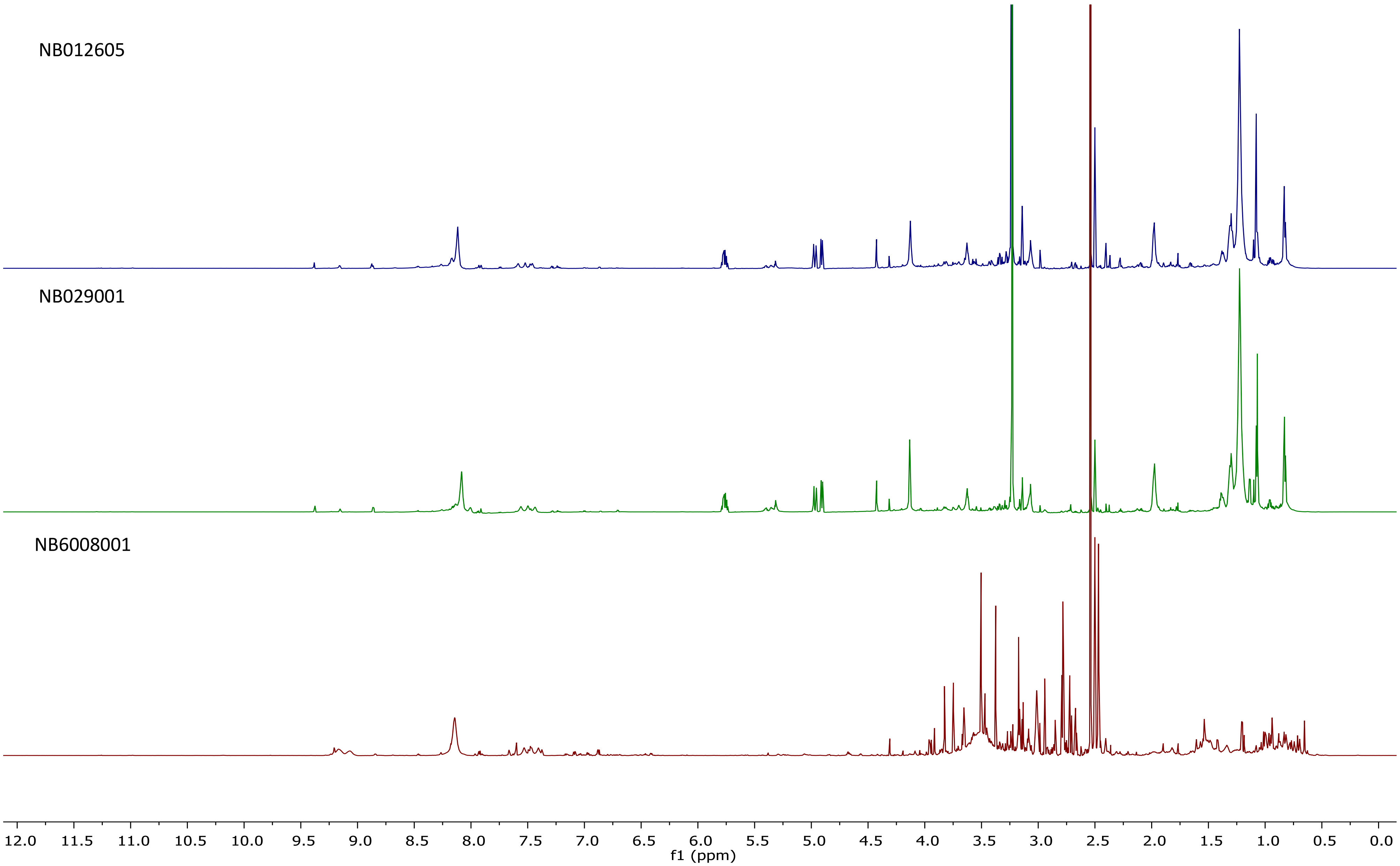

[a]Singlet at δ_H 2.54 represents DMSO residual peak

S5 ^{1}H NMR (800 MHz) Spectra of *Oceanapia* sp. Extracts in DMSO-d_6[a]

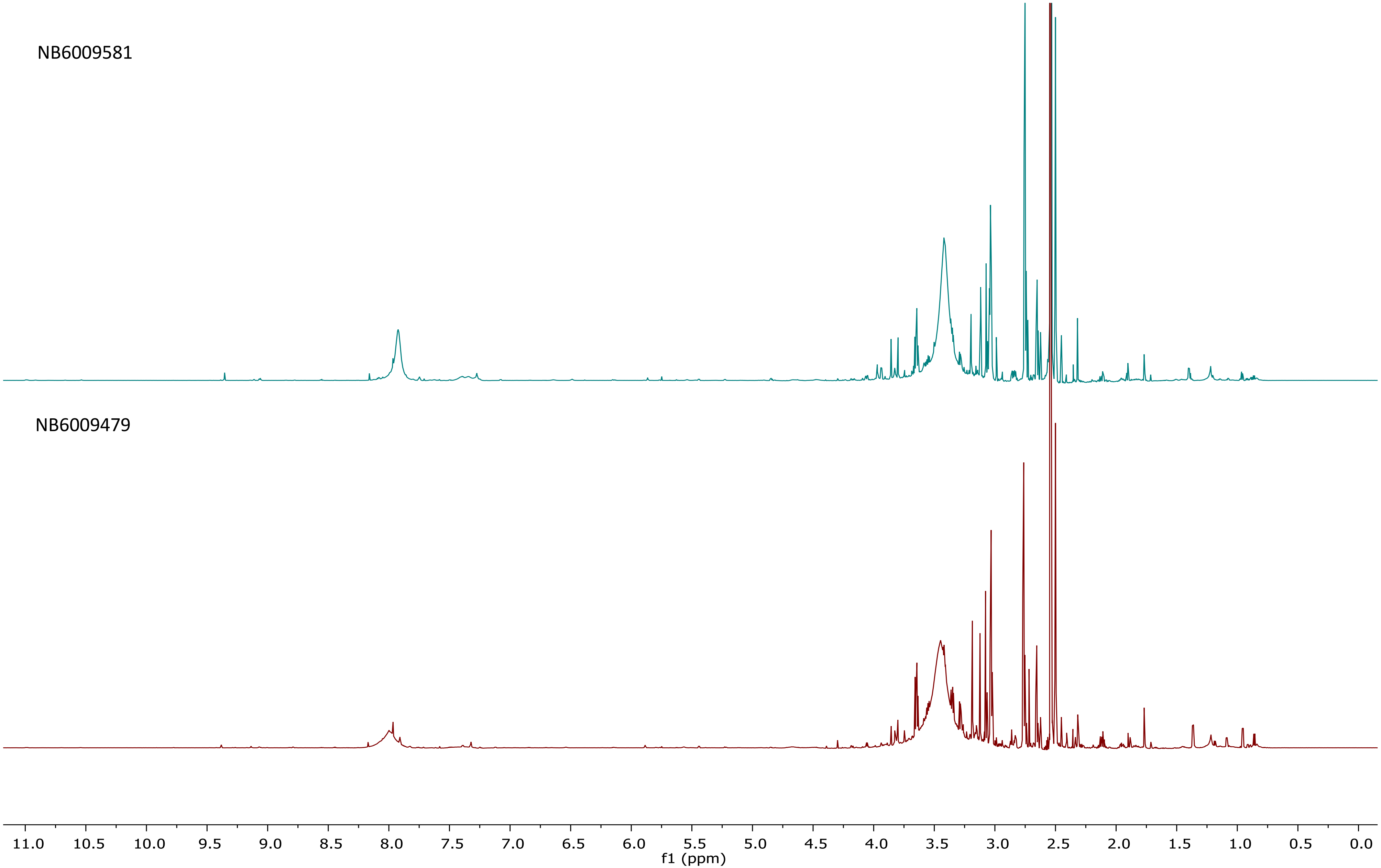

[a]Singlet at δ_H 2.54 represents DMSO residual peak

S6 ^{1}H NMR (800 MHz) Spectra of *Phyllospongia* sp. Extracts in DMSO-d_6[a]

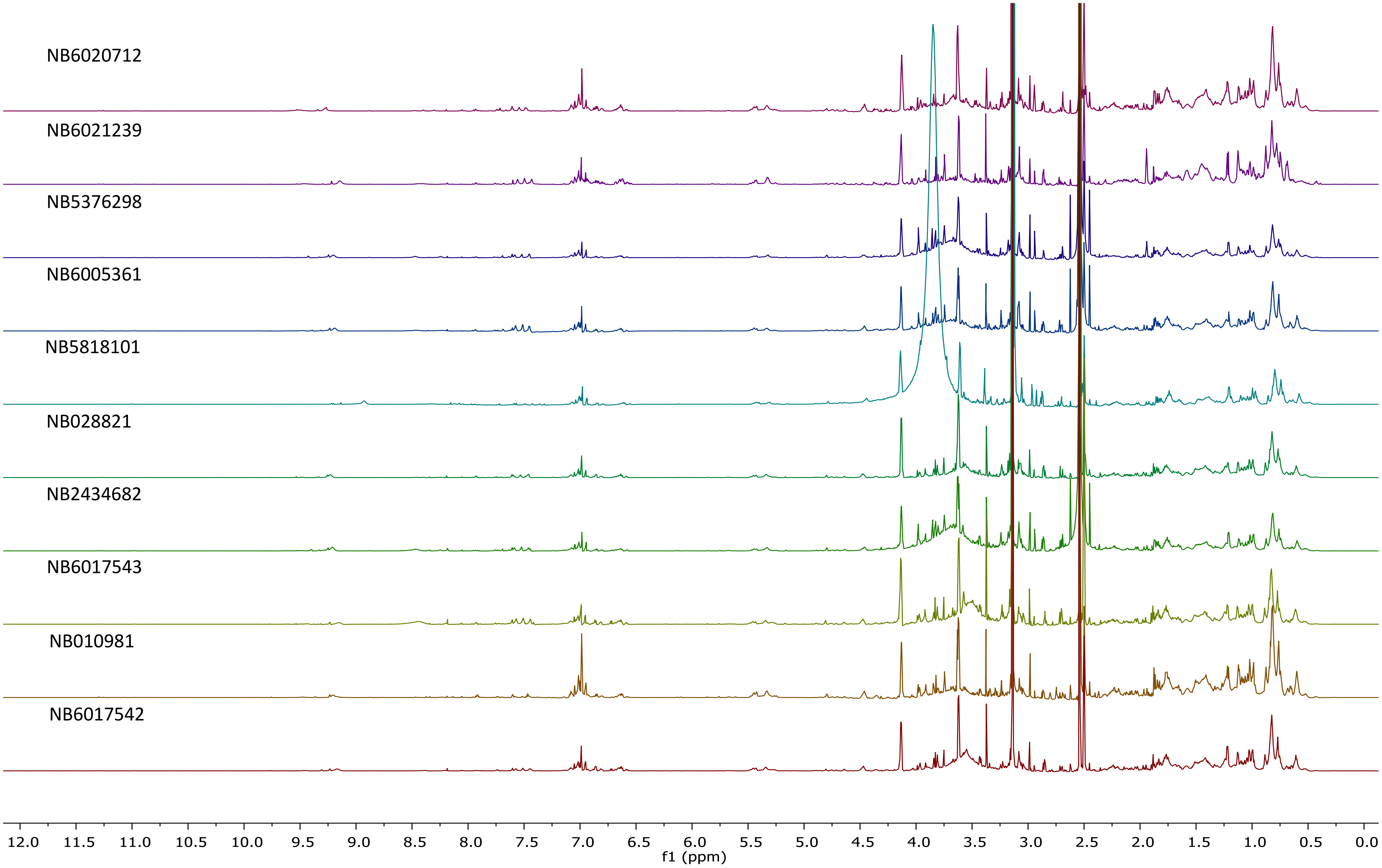

[a]Singlet at δ_H 2.54 represents DMSO residual peak

S7 ^{1}H NMR (800 MHz) Spectra of *Psammocinia* sp. Extracts in DMSO-d_6[a]

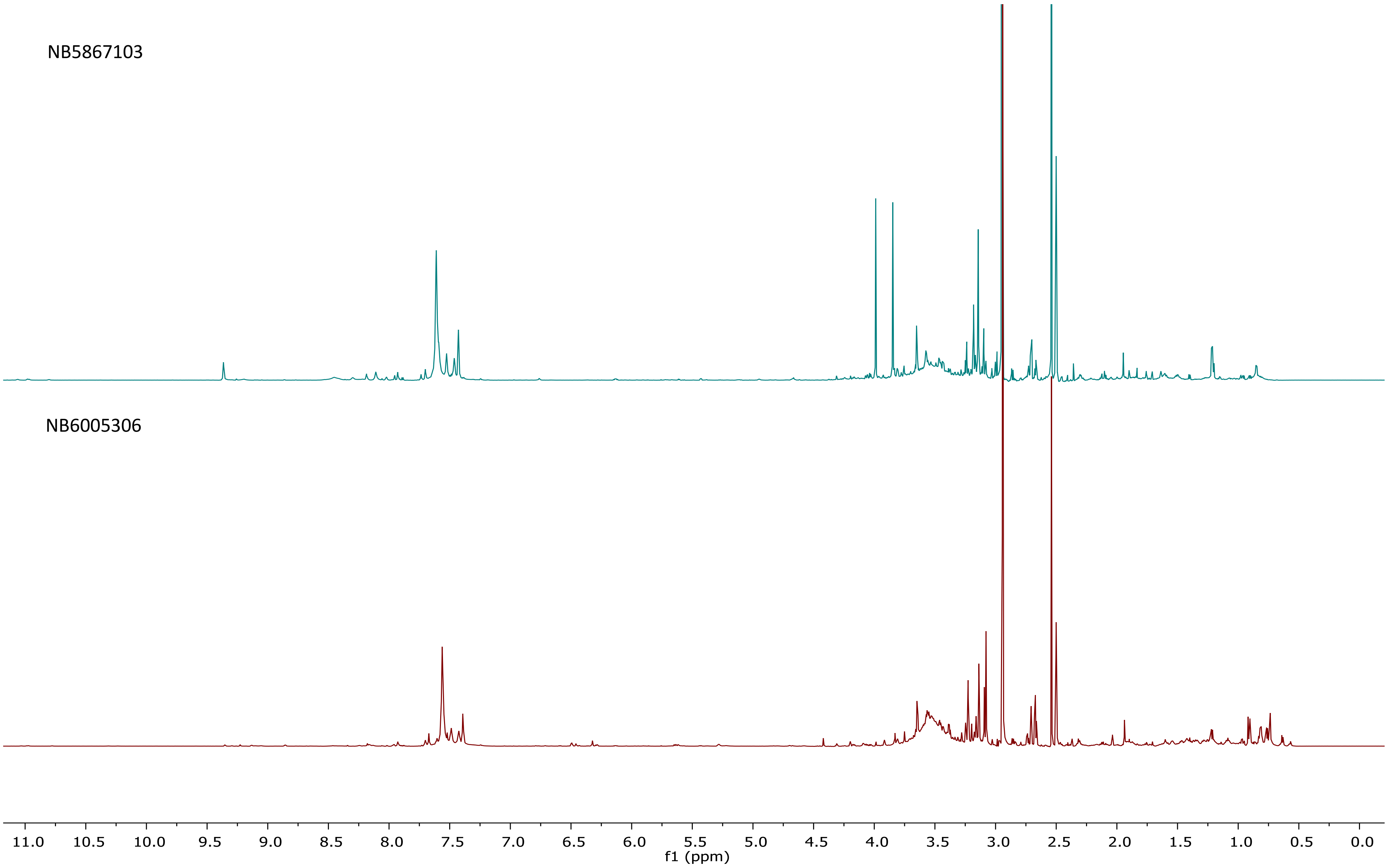

[a]Singlet at δ_H 2.54 represents DMSO residual peak

S8 ^{1}H NMR (800 MHz) Spectra of *Rhabdastrella globostellata* Extracts in DMSO-d_6[a]

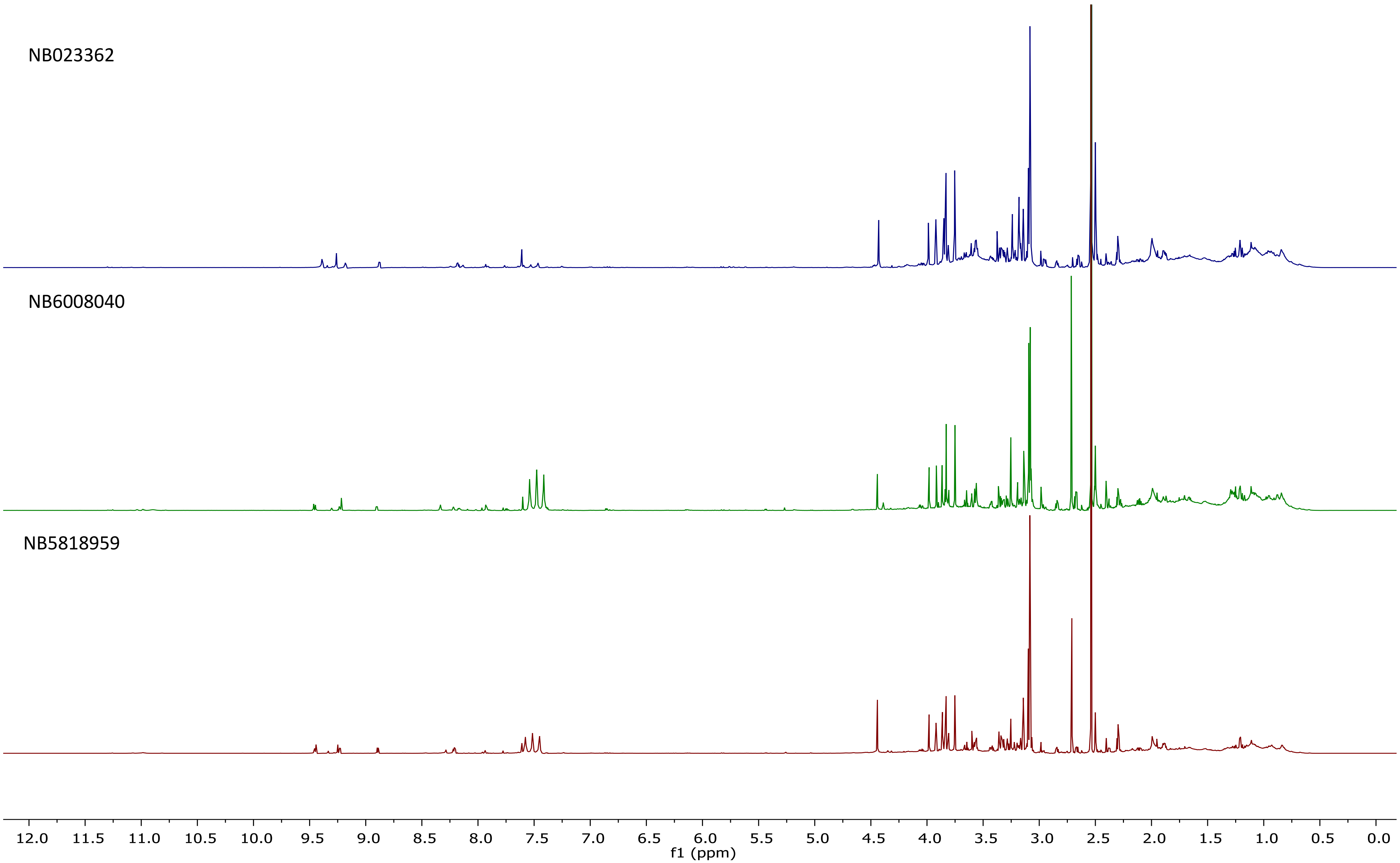

[a]Singlet at δ_H 2.54 represents DMSO residual peak

S9 ^{1}H NMR (800 MHz) Spectra of *Didemnum* sp. Extracts in DMSO-d_6[a]

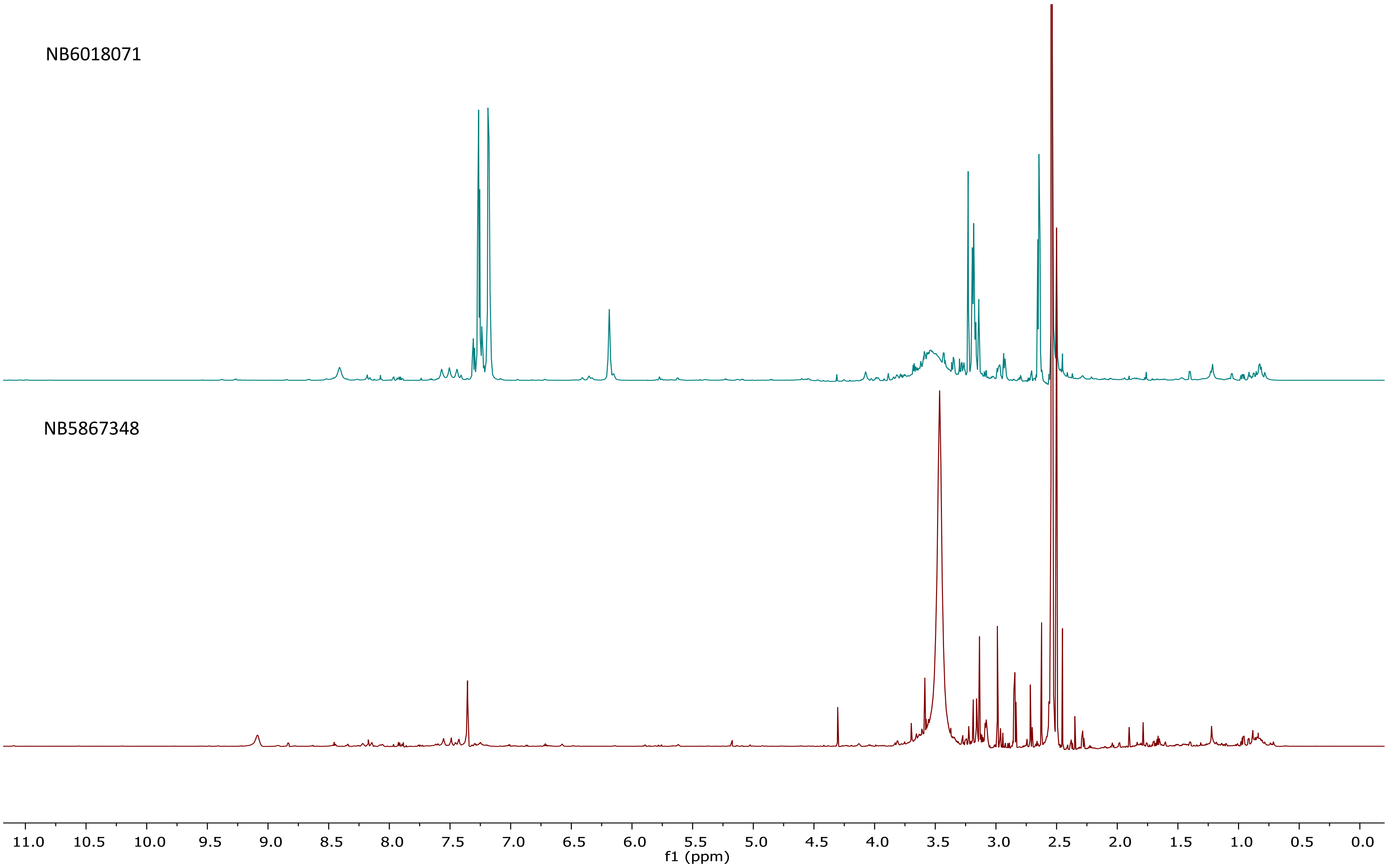

[a]Singlet at δ_H 2.54 represents DMSO residual peak

S10 ^{1}H NMR (800 MHz) Spectra of *Acanthophora muscoides*, *Agelas axifera*, *Ceratopsion clavatum*, *Chalinula* sp. and *Cinachyrella (Raphidotethya) enigmatica* Extracts in DMSO-d_6[a]

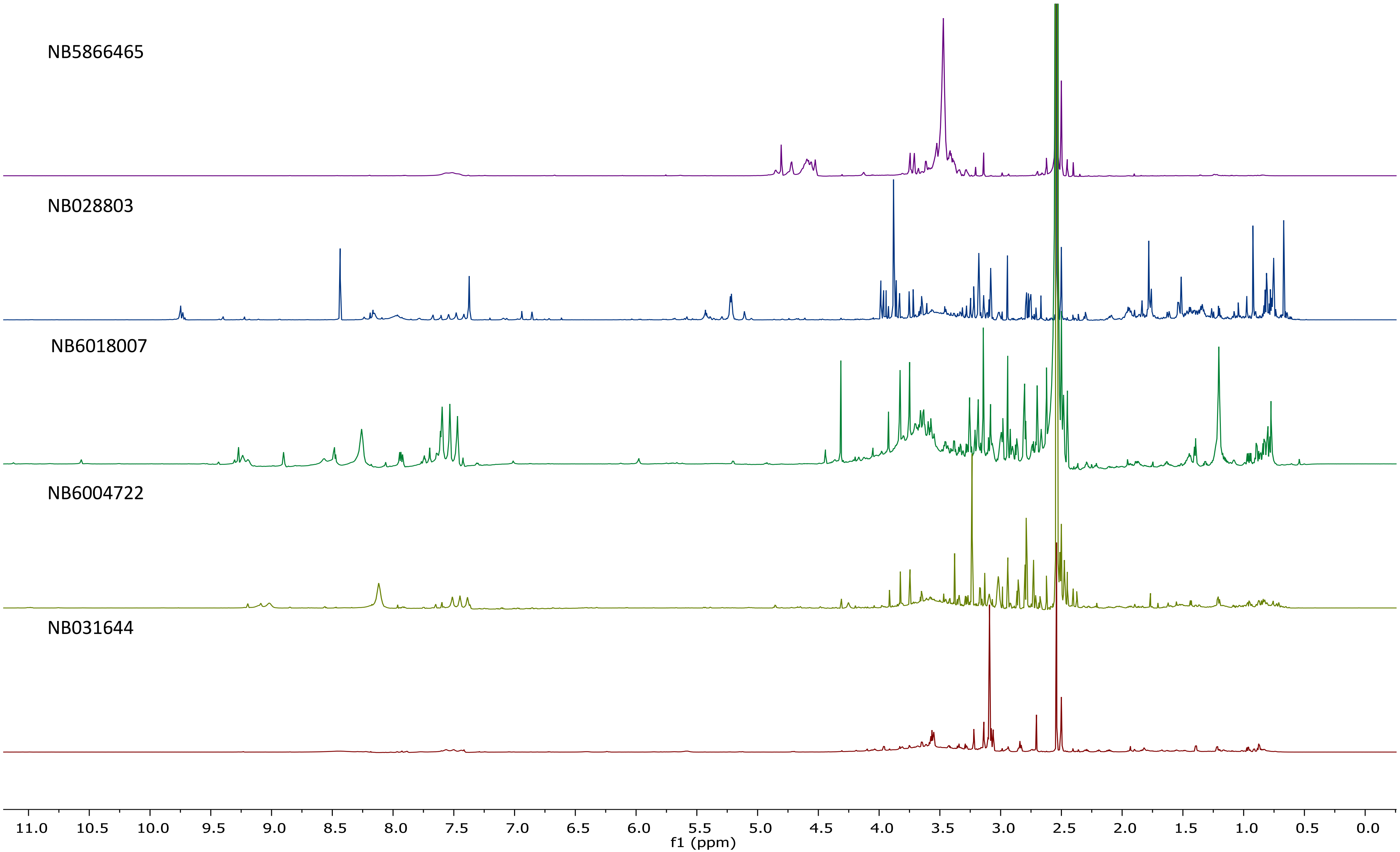

[a]Singlet at δ_H 2.54 represents DMSO residual peak

S11 ^{1}H NMR (800 MHz) Spectra of *Citronia* sp., *Cymbastela coralliophila*, *Desmacella* sp., *Echinochalina (Protophlitaspongia)* sp. and *Endectyon* sp. Extracts in DMSO-d_6[a]

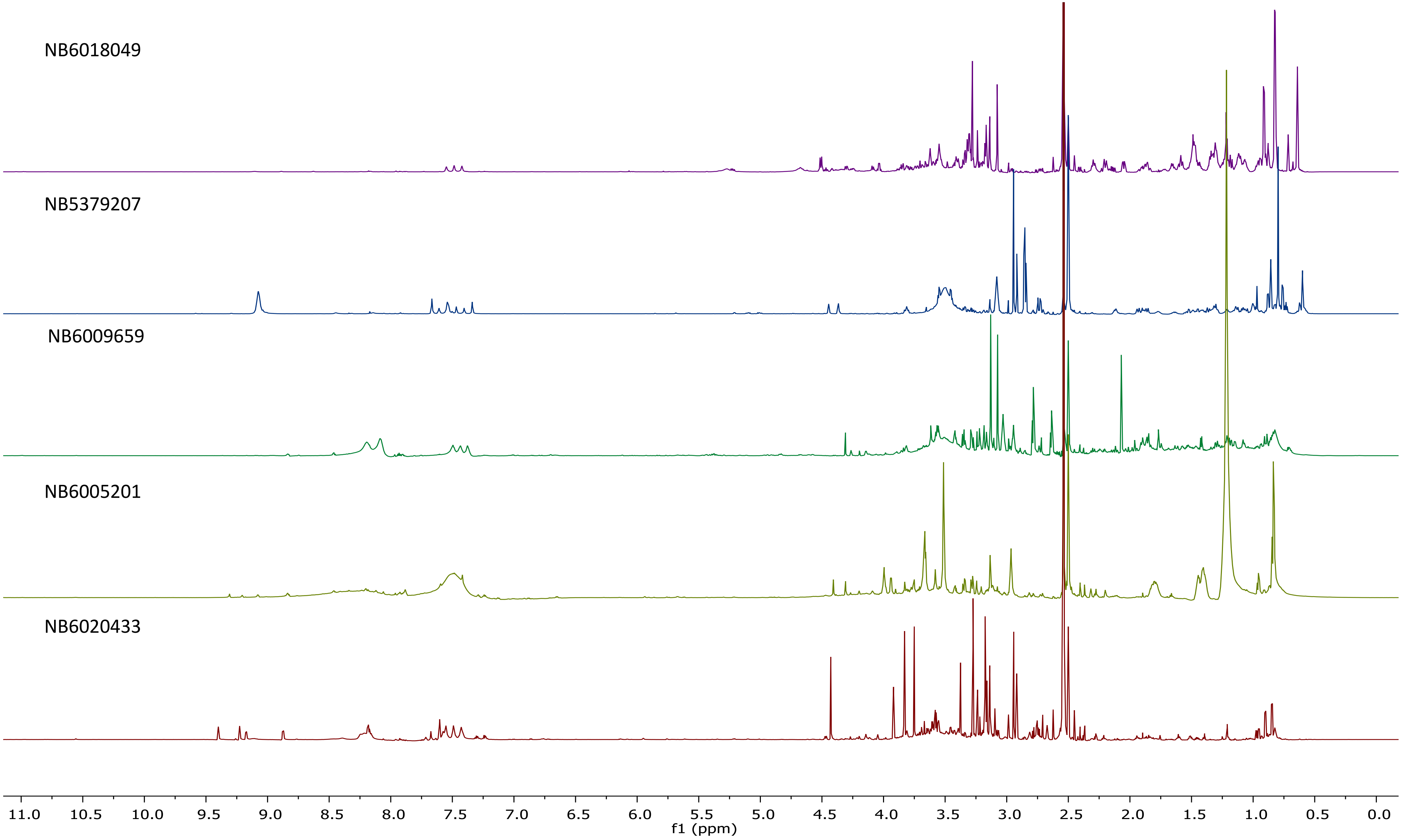

[a]Singlet at δ_H 2.54 represents DMSO residual peak

S12 ^{1}H NMR (800 MHz) Spectra of *Erylus amissus*, *Fascaplysinopsis reticulata*, *Gelliodes* sp., *Ianthella flabelliformis* and *Leucetta microraphis* Extracts in DMSO-d_6[a]

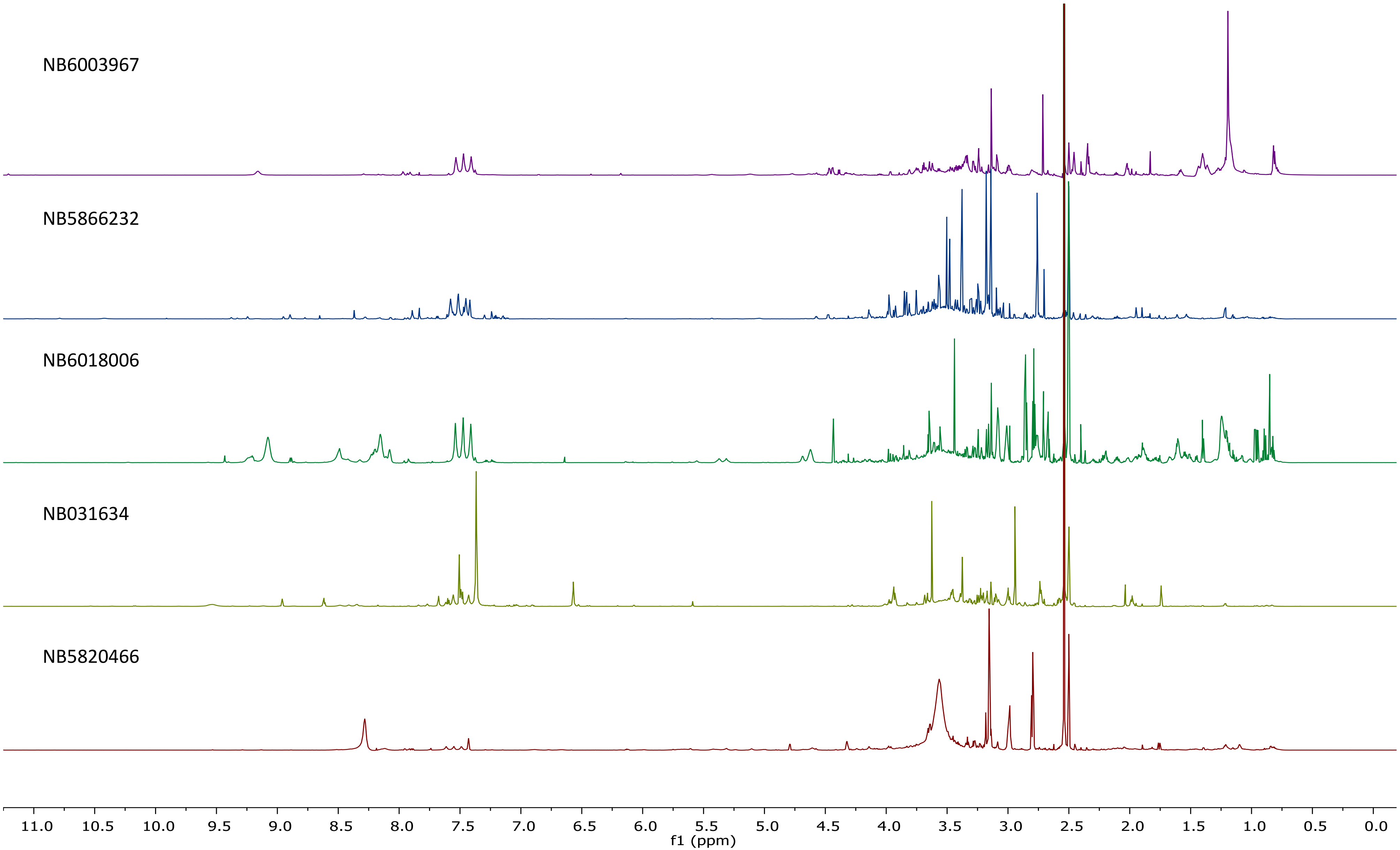

[a]Singlet at δ_H 2.54 represents DMSO residual peak

S13 ^{1}H NMR (800 MHz) Spectra of *Petromica (Chaladesma) pacifica*, *Polyfibrospongia flabellifera*, *Polycarpa aurata* and *Sarcophyton cherbonnieri* Extracts in DMSO-d_6[a]

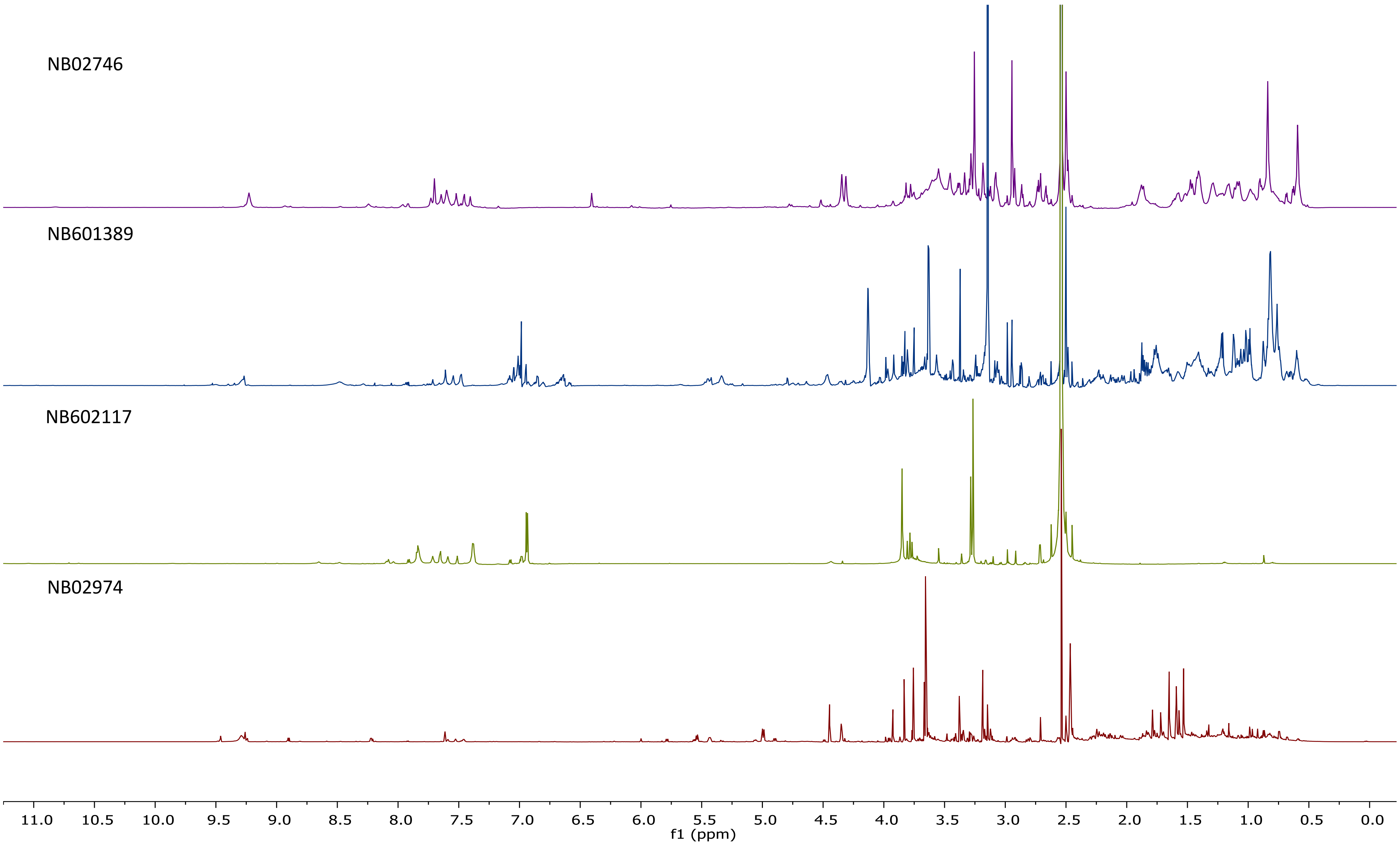

[a]Singlet at δ_H 2.54 represents DMSO residual peak
